# Hospital Meetings

**Published:** 1906-02-03

**Authors:** 


### HOSPITAL MEETINGS.

### THE GERMAN HOSPITAL.

There was but a small attendance at the annual general meeting of the Governors of the German Hospital, held at Winchester House on January 30.

The chair was taken by Mr. Arthur J. Allen, who, in moving the adoption of the annual report, said that it contained an extremely satisfactory account of the doings of the hospital during the last year. He was glad to be able to make the gratifying announcement that H.R.H. the Duke of Connaught had consented to become President of that hospital in succession to H.R.H. the Duke of Cambridge, and had also kindly promised to preside at their anniversary festival, to be held next May. They were very much indebted to their Treasurer, Baron Schröder for his efforts in this direction. They sincerely hoped that, large as was the sum collected at their last festival, this year's amount would exceed it.

There had been a slight outbreak of scarlet fever in the hospital during the past year, but, fortunately, it was only of a mild type. It was the first outbreak they had had since the hospital was opened. They were glad to note that the donation from King Edward's Hospital Fund showed an increase this year, being £200, as against £150 in 1904. They hoped to receive a still larger amount in the future.

It was a cause of great gratification to the committee to find that they would receive £2,500 from the "Bawden Fund," and for this they must thank Mr. Edgar Speyer, in whose hands the apportionment of the fund had been left.

The report having been adopted, the customary votes of thanks were passed, and the proceedings then terminated.

